# Twin Anemia-Polycythemia Sequence With Spontaneous Resolution in Dichorionic Diamniotic Twin Pregnancy: A Case Report and a Review of the Literature

**DOI:** 10.7759/cureus.73563

**Published:** 2024-11-12

**Authors:** Yoshihiro Yoshimura, Katsusuke Ozawa, HIroyuki Goto, Yu Yamazaki, Hitoshi Isohata, Daigo Ochiai

**Affiliations:** 1 Obstetrics and Gynecology, Kitasato University School of Medicine, Sagamihara, JPN; 2 Center for Maternal-Fetal, Neonatal and Reproductive Medicine, National Center for Child Health and Development, Tokyo, JPN

**Keywords:** dichorionic diamniotic twin, placenta, prenatal diagnosis, twin anemia polycythemia sequence, ultrasound

## Abstract

Complications of twin pregnancy such as twin anemia-polycythemia sequence (TAPS), which involve placental anastomotic vessels, occur mostly in monochorionic diamniotic twins and they have rarely been reported in dichorionic diamniotic (DD) twins. Here, we report a case of DD twins diagnosed with TAPS with fetal hydrops caused by fetal anemia at 28 weeks, which resolved spontaneously during pregnancy.

A 37-year-old pregnant woman was referred to our hospital because of a twin pregnancy at 13 weeks. The fetus showed the lambda sign, leading to the diagnosis of DD twin. At 28 weeks, fetal ascites and subcutaneous edema were observed in twin A. In blood flow assessment, elevated middle cerebral artery peak systolic velocity (MCA-PSV) 88.0 cm/s (2.3 MoM), pulsatile flow in the umbilical vein, and increased flow in the ductus venosus were also found, but no abnormal flow in the umbilical artery was found in twin A. In contrast, there were no signs of hydrops and MCA-PSV was 38.7 cm/s (1.0 MoM) in twin B. Both fetuses did not show any abnormality of amniotic fluid volume. Thus, the twin was diagnosed with TAPS in DD twins. Fetal hydrops could be resolved spontaneously, and MCA-PSV gradually decreased. Two female infants weighing 2,366 g and 2,048 g were delivered by cesarean section at 35 weeks. Blood tests demonstrated Hb 8.5 g/dl and reticulocyte 7.4% in twin A and Hb 13.3 g/dl and reticulocyte 6.1% in twin B, respectively.

This case highlights that complications of twin pregnancy involving placental anastomotic vessels such as TAPS could occur even in DD twins.

## Introduction

In twin pregnancies, vascular anastomosis of the placentas allows the transfer of blood from one fetus. Disproportionate transfusions between both fetuses can lead to a variety of complications such as twin-twin transfusion syndrome (TTTS) and twin anemia-polycythemia sequence (TAPS).

TAPS causes a large difference in hemoglobin concentration in both infants due to the slow shunt blood flow through the vascular anastomosis from the donor to the recipient, resulting in anemia in the donor and polycythemia in the recipient without an abnormality of amniotic fluid volume. TAPS may occur spontaneously or after laser surgery for TTTS. The spontaneous form complicates approximately 3-5% of monochorionic twin pregnancies whereas the post-laser form occurs in 2-13% of TTTS cases [1-3].

Complication characteristics of twin pregnancy involving placental anastomotic vessels occur mostly in monochorionic diamniotic (MD) twins, and they have rarely been reported in dichorionic diamniotic (DD) twins. Here, we report a case of DD twins diagnosed with TAPS with fetal hydrops caused by fetal anemia at 28 weeks of gestation, which resolved spontaneously during pregnancy.

## Case presentation

**Figure 1:**
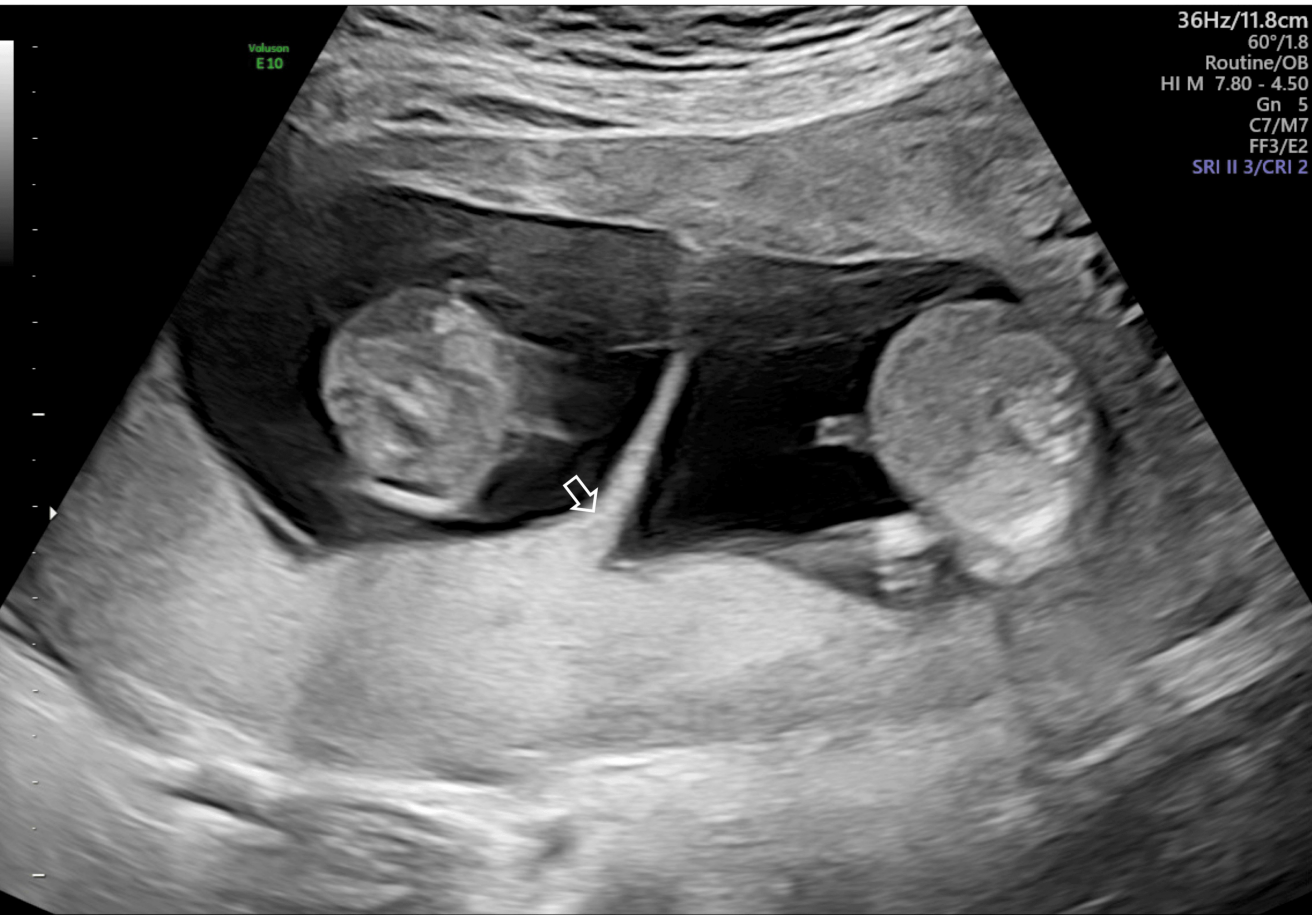
Fetal ultrasound findings at 13 weeks The fetus showed the lambda sign (arrow), resulting in the diagnosis of dichorionic diamniotic twin.

A 37-year-old pregnant woman, 1-para, was referred to our hospital because of a twin pregnancy at 13 weeks of gestation. On ultrasound (US) examination, the fetus showed appropriate growth for its gestational age and the lambda sign, resulting in the diagnosis of DD twin (Figure 1).

**Figure 2:**
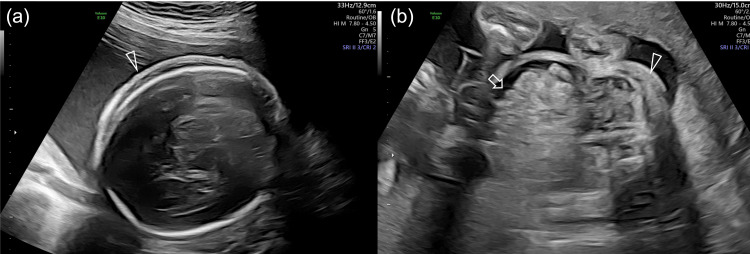
Fetal ultrasound findings at 28 weeks in twin A Subcutaneous edema (arrowhead) (a, b) and fetal ascites (arrow) (b) were observed.

**Figure 3:**
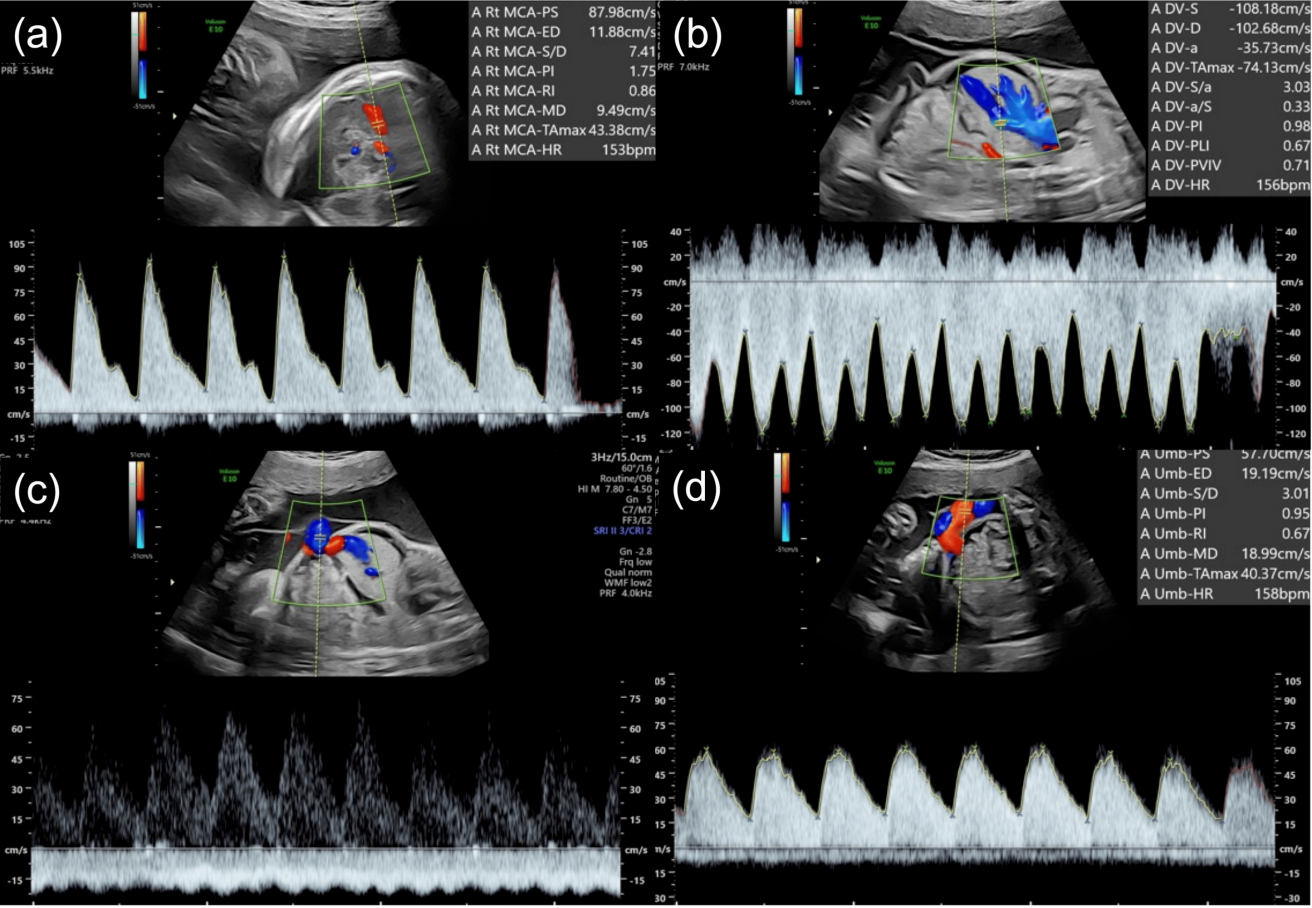
Fetal blood flow assessment at 28 weeks in twin A Elevated middle cerebral artery peak systolic velocity of 87.9 cm/s (2.3 MoM) (a), increased flow in the ductus venosus (b), and pulsatile flow in the umbilical vein (c) were observed, but no abnormal flow was observed in the umbilical artery (d).

On the fetal US at 28^5/7^ weeks of gestation, an estimated fetal weight of 1197ｇ(-0.4 SD), a maximal vertical pocket of 6.0 cm, fetal ascites, and subcutaneous edema were observed in twin A (Figure 2). In blood flow assessment, elevated middle cerebral artery peak systolic velocity (MCA-PSV) of 88.0 cm/s (2.3 MoM), pulsatile flow in the umbilical vein, and increased flow in the ductus venosus were also found, but no abnormal flow in the umbilical artery was found in twin A (Figure 3). 

**Figure 4:**
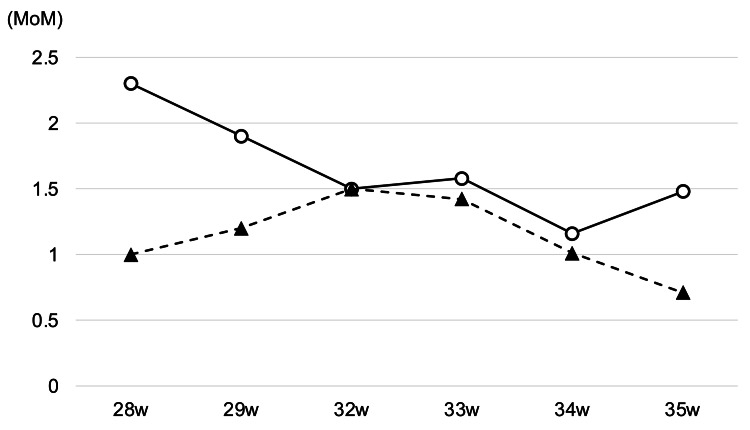
Changes in middle cerebral artery peak systolic velocity values in pregnancy Solid line: twin A; dashed line: twin B.

In contrast, an estimated fetal weight of 1149 g (-0.6 SD), a maximal vertical pocket of 4.8 cm, no fetal ascites, no subcutaneous edema, and MCA-PSV of 38.7 cm/s (1.0 MoM) were observed in twin B. No morphological malformations were detected in either fetus. Maternal blood tests were found to be negative for irregular antibodies, HbF was 0.4 %, parvovirus B19 IgM was negative, and cytomegalovirus IgM was negative, indicating that no maternal factors could induce fetal anemia. Based on these findings, the twin was diagnosed with TAPS, with twin A being the TAPS donor and twin B being the TAPS recipient twin. Given the complexity of the clinical problem, we recommended a second opinion from Dr. Ozawa, a specialist in fetal treatment, for the patient due to the complexity of the clinical problem. Fetal hydrops in twin A could be spontaneously resolved at 29 weeks of gestation, and MCA-PSV gradually decreased and was less than 1.5 MoM after 34 weeks of gestation (Figure 4).

We performed an emergency cesarean section at 35^6/7^ weeks of gestation due to the onset of labor pain and delivered two female infants weighing 2,366 g with Apgar scores of 6 and 7 at 1 and 5 min and an umbilical artery pH of 7.28 (twin A) and 2,048 g with Apgar scores of 8 and 9 at 1 and 5 min and an umbilical artery pH of 7.25 (twin B), respectively. Blood tests demonstrated Hb 8.5 g/dl and reticulocyte 7.4% in twin A and Hb 13.3 g/dl and reticulocyte 6.1% in twin B, respectively. 

**Figure 5:**
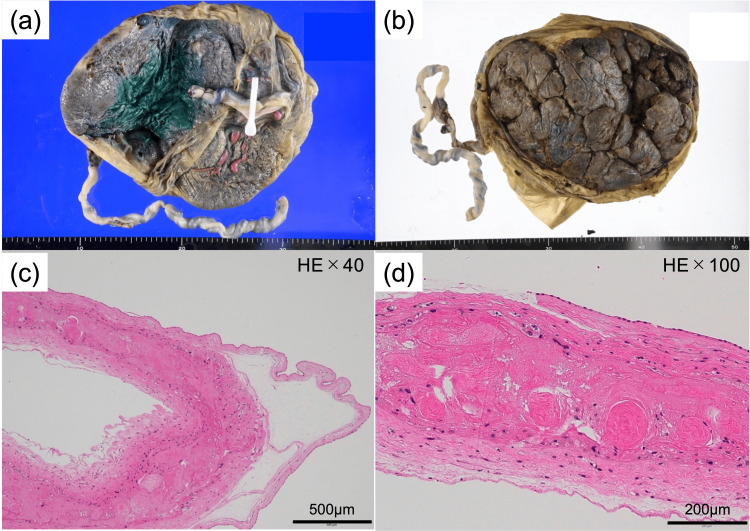
Placental findings Macroscopic findings of the placenta (a: fetal plane, b: maternal plane). HE staining of placental tissue (c: × 40, d: × 100).

Macroscopically, the placentas of both infants were fused (Figure 5a, 5b), and the dye injection test showed no obvious anastomotic vessels on the placental surface. Histopathological examination of the placenta confirmed the presence of two amniotic and two chorionic membranes (Figure 5c, 5d), leading to the diagnosis of DD placenta.

After birth, the donor required oral iron therapy for anemia but did not require a blood transfusion. She was discharged at the age of 23 days. The oral iron supplementation was completed at the age of eight months. The recipient was treated with continuous positive airway pressure for neonatal transient hypercapnia until day 3, and she was discharged at the age of 37 days. Now, both infants grow normally and have normal mental development at the age of eight months.

## Discussion

To date, there is limited information about pregnancies complicated by TAPS in DD twins. In our case, severe anemia and fetal hydrops were detected without abnormality of amniotic fluid volume in one of the twins. These symptoms gradually resolved without any therapeutic intervention. Meanwhile, the other twin did not show fetal hydrops or abnormality of amniotic fluid volume, and the MCA-PSV ranged between 1.5 MoM and 0.7 MoM. 

The prenatal diagnosis of TAPS is based on the finding of discordant MCA Doppler abnormalities, including MCA-PSV >1.5 MoM in the donor with anemia and MCA-PSV <1.0 MoM in the recipient with polycythemia [4]. In addition, TAPS is prenatally classified according to whether there is a cardiac compromise in the donor (absent or reversed end-diastolic flow in the umbilical artery, pulsatile flow in the umbilical vein, or increased or reversed flow in the ductus venosus), hydrops of the donor, or death of one or both fetuses [4]. Based on the definition, our case is categorized into stage 4 in antenatal TAP classification due to fetal hydrops. MCA-PSVs of our twins were 2.3 MoM and 1.0 MoM when fetal anemia was noted. The donor fetus met the TAPS criteria with MCA-PSV >1.5. In contrast, when strictly considered, the recipient did not meet the criteria for MCA-PSV <1.0 MoM. The difference in MCA-PSV between fetuses is 1.3 MoM, which is over the optimal threshold for MCA-PSV discordance of ≥1.0 MoM agreed with over 80% of the experts [5]. Thus, we assume that the phenomena that occurred in our case at 28 weeks of gestation can be interpreted as the pathophysiology of TAPS.

The heterogeneity of the disease leads to a broad range of outcomes in TAPS. There are several treatment options for TAPS, including expectant management, induction of labor, and intrauterine blood transfusion [3]. In previous reports, evident US signs of TAPS in MD twins persisted for several weeks but eventually resolved without any therapeutic intervention [6] and they hypothesized that spontaneous resolution likely resulted from spontaneous thrombosis of the residual vascular anastomosis [6]. In our case, TAPS occurred in DD twins at 28 weeks of gestation, and symptoms gradually resolved spontaneously after 29 weeks of gestation. Consequently, the hemoglobin difference and the reticulocyte ratio between the twins after birth did not meet the diagnostic postnatal criteria for a hemoglobin difference of >8.0 g/dl and a reticulocyte ratio of >1.7 in TAPS. TAPS is thought to result mainly from slow intertwin blood transfusion through small arteriovenous anastomoses (<1 mm) [6]. In our case, the dye injection test of the placenta showed no obvious anastomotic vessels on the placental surface and placental histology did not detect the presence of vascular anastomoses and microthrombi in the deep placenta. These placental findings also supported the diagnosis of TAPS. 

There are limited reports of complications characteristic of twin pregnancy resulting from vascular anastomosis in the placenta in DD twins such as TAPS [7-12], TTTS [13-18], and TRAP sequence [19]. Although six cases of TAPS in DD twins have been reported [7-12] (Table 1), TAPS was diagnosed prenatally in only three of the six cases. Similar to our case, the timing of the TAPS diagnosis was at 27, 31, and 32 weeks of gestation, respectively. Of these cases, intrauterine blood transfusion was performed in two cases, and cesarean delivery was selected immediately after the onset of TAPS in one case. The postnatal hemoglobin difference between the twins ranged from 12.6 to 21.7 g/dl, and none of the cases resolved spontaneously during pregnancy, except ours. Thus, TAPS could occur even in DD twins. In such cases, the clinical course may be as varied as in cases of MD twins.

**Table 1:** A literature review on reported cases complicated with twin anemia-polycythemia sequence in dichorionic diamniotic twins GW: gestational weeks; MCA-PSV dif: the difference in MCA-PSV; Hb dif: the difference in Hb; ND: not determined; IUT: intrauterine blood transfusion; CS: cesarean section; R: recipient; BTF: blood transfusion.

Case	Age	GW	MCA-PSV dif (MoM)	Treatment	Hb dif (g/dl)	Reticulocyte ratio	Anastomosis	Fetal outcome
Jeyaseelan et al. [7]	31	27	1.3	IUT, 29w CS	19.3	ND	-	R: BTF
Zilliox et al. [8]	35	31	1.3	IUT, 31w CS	18.5	3.3	+	R: Dead 24 h after birth
Harada et al. [9]	23	31	ND	31w CS	18.6	5	-	R: BTF
Kanagaretnam et al. [10]	34	32	0.7	32w CS	21.7	ND	ND	R: BTF
Tollenaar et al. [11]	22	33	ND	33w CS	12.6	ND	+	R: BTF
Lee et al. [12]	36	33	ND	33w CS	14.4	ND	-	R: BTF
Our case	37	28	1.3	Observation, 35w CS	4.8	1.2	-	No BTF

## Conclusions

This case highlights that complications of twin pregnancy involving placental anastomotic vessels could occur in DD twins. TAPS and TTTS should be considered in DD twins, especially when two placentas are located in contact with each other. Similar to MD twins, antenatal TAPS in DD twins may resolve spontaneously. Given the variety of outcomes in MD-TAPS cases, several treatment options should be considered in DD-TAPS cases from close monitoring to intrauterine blood transfusion.
